# Association between Hypometabolism in the Supplementary Motor Area and Fear of Falling in Older Adults

**DOI:** 10.3389/fnagi.2017.00251

**Published:** 2017-07-28

**Authors:** Ryota Sakurai, Yoshinori Fujiwara, Masashi Yasunaga, Hiroyuki Suzuki, Kazuyuki Kanosue, Manuel Montero-Odasso, Kenji Ishii

**Affiliations:** ^1^Research Team for Social Participation and Community Health, Tokyo Metropolitan Institute of Gerontology Tokyo, Japan; ^2^Faculty of Sport Sciences, Waseda University Saitama, Japan; ^3^Japan Society for the Promotion of Science Tokyo, Japan; ^4^Gait and Brain Laboratory, Parkwood Institute, University of Western Ontario and Lawson Health Research Institute London, ON, Canada; ^5^Research Team for Neuroimaging, Tokyo Metropolitan Institute of Gerontology Tokyo, Japan

**Keywords:** fear of falling, supplementary motor area, aging brain, older adults, longitudinal study

## Abstract

**Background**: A better understanding of the neural mechanisms that underlie the development of fear of falling (FoF) in seniors may help to detect potential treatable factors and reduce future falls. We therefore investigate the neural correlates of FoF in older adults using ^18^F-fluorodeoxyglucose-positron emission tomography (FDG-PET).

**Methods**: This cohort study included 117 community-dwelling older adults. At baseline, participants were assessed for FoF, psychiatric symptoms, walking speed, global cognition and cerebral glucose metabolism with FDG-PET. The incidence of FoF in the participants who did not report FoF (N-FoF) at baseline was again ascertained 2 years later. FDG uptake was compared between the FoF and non-FoF groups. Logistic regression analyses to examine the predictors of newly developed FoF (D-FoF) using normalized regional FDG uptake were then performed.

**Results**: At baseline, 50.4% (*n* = 59) of participants had FoF. The FoF group had significantly decreased glucose metabolism in the left superior frontal gyrus (supplementary motor area, SMA; BA6) compared to the non-FoF group. After 2 years, 19 out of the 58 participants in the non-FoF group developed FoF. Logistic regression analysis revealed that decreased cerebral glucose metabolism in the left SMA at the baseline was a significant predictor of the future development of FoF, independently of psychiatric symptoms and walking speed.

**Conclusion**: In healthy older adults, hypometabolism in the left SMA, which is involved in motor planning and motor coordination, contributes to the development of FoF. Our result might help elucidate underlying mechanism of the association between deficits in motor control and FoF.

## Introduction

Fear of Falling (FoF) is a clinical entity that includes symptoms related to fall-related fear, concern about falling and lack of balance confidence. The prevalence of FoF varies between 21% and 85% and is higher in women (Arfken et al., 1994; Niino et al., 2000; Scheffer et al., 2008). FoF is a major mental health problem with potentially serious outcomes for older adults. Numerous studies have indicated that FoF is both a risk factor and consequence of impaired physical function and falls (Arfken et al., 1994; Cumming et al., 2000; Friedman et al., 2002; Lach, 2005; Austin et al., 2007; Scheffer et al., 2008; Denkinger et al., 2015). Thus, a better understanding of the neural mechanisms that underlie the development of FoF in older adults without physical disabilities may help to detect potential treatable factors and reduce future falls.

The finding of previous studies raised the possibility that deficits in motor control, including increased gait variability, might directly cause or worsen FoF by increasing gait unsteadiness (Rochat et al., 2010; Ayoubi et al., 2014; Sawa et al., 2014). Indeed, recent systematic review supports this hypothesis (Ayoubi et al., 2015). Motor control, which is involved in regulating gait variability, relies on frontal brain networks which are vulnerable to the aging process and comorbidities (Raz et al., 1997, 2004; Seidler et al., 2010). Therefore, disruptions of these networks including reduced neural activity in the frontal lobe may pose a risk of new-onset of FoF through impairment of motor control.

This hypothesis of the association of frontal brain networks with FoF is partly supported by the results of a study by Tuerk et al. (2016), who reported that decreased regional gray matter volume in areas, such as the left supplementary motor area (SMA), was correlated with increased concern about falls. Furthermore, their results suggested that this relationship is primarily due to psychological factors and not physical factors, such as muscle strength and postural balance, because the significance of the relationships is diminished after adjusting for anxiety and neurotic personality. Although these results are consistent with the finding that FoF is also strongly affected by psychological deficits and personality (Delbaere et al., 2010; Painter et al., 2012), the neural basis of new-onset FoF is still unclear.

The goal of this study therefore was to elucidate the neural correlates of having and developing FoF in community-dwelling older adults. We hypothesize that new-onset FoF would have a stronger relationship with reduced neural activity in the frontal brain networks, and that this association would be explained by psychological factors as shown by the previous study. To this end, we conducted a 2-year follow-up study and used ^18^F-fluorodeoxyglucose-positron emission tomography (FDG-PET), which measures the cerebral metabolic rate of glucose as a proxy of neural activity. In the analysis consisting of participants with no FoF at baseline, the baseline differences in cerebral metabolism between participants who did not report FoF (N-FoF) and who had newly developed FoF (D-FoF) at the follow-up assessment were compared; then a logistic regression analysis was conducted to examine the independent predictors of the presence of newly developed FoF at the follow-up assessment using the regional metabolic value of the brain areas with statistically significant differences in the aforementioned voxel-wise analysis.

## Materials and Methods

### Participants

One hundred and seventeen community-dwelling older adults (mean ± standard deviation age, 74.0 ± 5.3 years; women, 77.8%) were recruited from the Tokyo Metropolitan Institute of Gerontology (TMIG) database to participate in the present study. Participants were included if they met the following criteria: (i) 60 years old or older; (ii) stable medical condition; (iii) fully functional in terms of instrumental activities of daily living (ADL), which were assessed with the TMIG-Index of Competence (TMIG-IC; Koyano et al., 1991); and (iv) willing to consent to a FDG-PET assessment. The exclusion criteria included the following: (i) presence of any neuromuscular and/or mental disorder; (ii) history of a cerebrovascular disorder; (iii) diagnosis of parkinsonism by an experienced physician’s interview (YF); (iv) gait disturbances, such as walking aid-dependent; (v) use of psychoactive medications or tranquilizers; and (vi) diagnosis of anatomical abnormalities, such as high levels of cortical atrophy or grade-3 (Fazekas scale) white matter hyperintensities (WMH), on magnetic resonance imaging (MRI) by an experienced neuroradiologist (KI). During enrollment, no participants reported episodes of dizziness, tinnitus, or significant hearing loss.

The study was conducted in accordance with the ethical standards of the Declaration of Helsinki. The research protocol was approved by the TMIG. All participants gave written informed consent.

### Measures

The participants underwent comprehensive assessments, including medical and psychological interviews and physical and cognitive evaluations, within 3 months of the FDG-PET scan. The comprehensive assessments were conducted in an ambulatory tertiary health center. Two years after the comprehensive baseline assessment, a follow-up assessment was conducted to determine if any participants who had not reported FoF at baseline had developed FoF during the follow-up period (mean, 23.5 months).

#### Fear of Falling

The presence of FoF was determined with a questionnaire widely used in previous studies (Maki et al., 1991; van Haastregt et al., 2008; Sakurai et al., 2017a). The participants were asked to respond “yes/no” to the question: “Are you afraid of falling?” They were assigned to the FoF or non-FoF group based on their responses.

#### Medical and Psychological Variables

All participants were interviewed by either a physician or physical therapist who assessed their health-related characteristics, including demographics, comorbidities, history of hospitalization, medication and body-mass index (BMI). Years of education, frequency of going outdoors, fall history in previous year, subjective health, functional capacity, depression symptoms, anxiety, neuroticism and self-esteem were also assessed by a trained interviewer. The frequency of going outdoors was defined as high (going out daily) or low (going out every few days a week or less). Fall was defined as an event resulting in an individual inadvertently coming to rest on the ground, floor or other lower level. Subjective health was assessed as excellent, good, fair, or poor, and the participants were assigned to either a good (excellent or good) or poor (fair or poor) group (Sakurai et al., 2014a). Functional capacity was evaluated with the TMIG-IC, which is a questionnaire consisting of three multidimensional subscales: the instrumental ADL, intellectual activity and social function, with higher scores indicating greater functional capacity (Koyano et al., 1991). Depressive symptoms were assessed with a 15-item version of the Geriatric Depression Scale (GDS; Almeida and Almeida, 1999). Anxiety was determined based on a questionnaire comprising nine questions regarding anxiety about health, social interaction, economics, disasters and crime prevention with each item rated on a 4-point scale (0, “not at all concerned” to 3, “very concerned”; Kobayashi et al., 2011). Neurotic personality was assessed with one question of the 36-item Short-Form Health survey; the participants were asked “Were you very nervous in the previous month?” The answers were rated 0–4 (“no” to “always”; Fukuhara et al., 1998). To measure self-esteem, which is a crucial variable in one’s overall sense of worthiness as a person, we used the Rosenberg Self-Esteem Scale (RSES; Mimura and Griffiths, 2007).

#### Physical and Cognitive Assessments

To assess physical function, we measured maximum grip strength, gait velocity under usual and fast conditions, Timed Up and Go (TUG) and postural balance (Sakurai et al., 2013). The maximum grip strength of the dominant hand was measured using a handheld dynamometer. For gait velocity, the participants were asked to walk once along an 11-m straight walkway on a flat surface at their usual pace and then walk twice along the walkway at their fastest and safest pace possible. A trained tester measured their walking duration using a chronometer (INTERVALTIMER SVAE109, Seiko Watch Corporation, Tokyo, Japan (temporal precision of within ± 0.0012%)). Velocity was calculated at a steady state by including only the data for the five middle meters of the 11-m pathway using the measured time. For the TUG, the participants were asked to stand up from a chair, walk around a marker located 3 m away, and return to and sit on the chair as fast as possible. Postural balance was measured with the one-leg standing test, which measures how long the participants can stand on their non-dominant leg with their eyes open. Global cognitive function was assessed using the Mini-Mental State Examination (MMSE; Fujiwara et al., 2010).

#### MRI/PET Scanning Protocol and Image Processing

After more than 5 h of fasting, each participant underwent FDG-PET scanning at the TMIG for research purposes. The blood of the participants was drawn before the MRI/PET scanning, and an analysis was conducted with a sequential autoanalyzer.

Three-dimensional (3D) MRI images were collected to detect abnormal brain structure (1.5-T Sigma Excite scanner, GE Healthcare, Milwaukee, WI, USA). 3D FDG-PET imaging (PET scanner SET 2400W; Shimadzu Corporation, Kyoto, Japan) was subsequently performed to evaluate the regional cerebral glucose metabolic values. Forty-five min after an intravenous injection of FDG (approximately 150 MBq), a 6-min emission scan was conducted to create images with a 128 × 128 (transverse section) × 63 (slice) matrix size and 2.0 × 2.0 × 3.125-mm voxel size. Attenuation was corrected with a transmission scan using a ^68^Ga/^68^Ge source. During the tracer-accumulation phase, the participants remained supine, quiet and motionless in a dimly lit and quiet room with their eyes open and their ears unplugged. A total of 1–2 mL of venous blood was drawn twice, immediately before the intravenous FDG injection and 30 min after the injection, and the plasma glucose concentration was measured.

Basic image processing was conducted with Dr. View software (AJS, Tokyo, Japan) and Statistical Parametric Mapping, version 8 (SPM8; Wellcome Trust Center for Neuroscience, London, UK), which is implemented in MATLAB (version R2014a; The MathWorks, Inc., Natick, MA, USA). All 3D-FDG-PET images were anatomically normalized and resampled (XYZ matrix, 79 × 95 × 80 mm; voxel size, 2 × 2 × 2 mm) with a FDG template that was created from the FDG images of 15 physically, neurologically and psychiatrically healthy subjects. The images were smoothed with a 12-mm full-width half-maximum isotropic kernel.

The regions of interest were the brain areas with statistically significant differences between participants who did not report FoF and who had newly developed FoF at the follow-up assessment in the voxel-wise analysis (see “Data Analysis” Section). The regional cerebral glucose metabolic values were normalized to cerebellar glucose metabolic values because the cerebellum is relatively preserved in older adults, even in patients with Alzheimer’s disease (Kuntzelmann et al., 2013). These normalized values were defined as the normalized regional cerebral metabolic rates of glucose (Sakurai et al., 2014b, 2017b).

#### Data Analysis

Comparisons between the non-FoF and FoF groups were made with chi-square or two-sample *t*-tests. With SPM8, two-sample *t*-test was performed to detect voxels with decreased glucose metabolism in the FoF group compared with the non-FoF group. The *t*-test was adjusted for the demographic and biological covariates of FoF and cerebral glucose metabolism, including gender, age, BMI, and blood glucose levels and the resulting significant and marginally significant differences between the non-FoF and FoF groups. The set cluster size was greater than 50 voxels, and the initial cluster threshold for statistical significance was set to *p* < 0.001, uncorrected. Clusters were considered significant when the *p* values were below a cluster-corrected *p*_(family–wise error)_ = 0.05. The locations of the brain regions were transformed from Montreal Neurological Institute coordinates into Talairach standard brain coordinates.

For the participants with no FoF at baseline, the differences in the measurement variables, including FDG-PET, at baseline between the participants who did not report FoF (N-FoF group) and who had newly developed FoF (D-FoF group) at the follow-up assessment were compared in the same manner as the aforementioned analyses. We then conducted a logistic regression analysis to examine the independent predictors of the presence of newly developed FoF at the follow-up assessment. For the logistic regression analysis, we set values for the regions of interest for which the voxel-wise analyses found statistical significance as independent variables and confirmed the absence of multicollinearity between the variables (variance inflation factors <0.3). Statistical analyses, excluding the two-sample *t*-tests for FDG-PET, were conducted with IBM SPSS PC for Windows (version 20.0, IBM Corporation, Armonk, NY, USA). All tests were two tailed, and *p* values less than 0.05 were considered statistically significant.

## Results

A total of 59 participants (50.4%) had FoF at the baseline assessment. Table 1 presents the characteristics of the participants in the non-FoF and FoF groups. Overall, the participants had a normal gait velocity (mean usual gait velocity, 1.49 m/s) and global cognitive functioning (mean MMSE, 29.3), without any instrumental ADL disability (mean TMIG-IC, 12.5). The FoF group had significantly more females, a higher GDS score, higher anxiety, less years of education (marginal significance, *p* = 0.065), lower RSES, and lower grip strength compared with the non-FoF group.

**Table 1:** Characteristics and differences of measurements of the participants with fear of falling (FoF) and without FoF (non-FoF) at baseline.

Baseline Characteristic, mean (SD)	All participants	FoF	non-FoF	*p* value
	(*n* = 117)	(*n* = 59)	(*n* = 58)	
Female, *n* (%)	91 (77.8)	51 (86.4)	40 (69.0)	0.023
Age	74.0 (5.3)	73.3 (5.5)	74.6 (5.0)	0.173
BMI	22.2 (3.1)	22.0 (3.3)	22.4 (3.0)	0.460
Years of education	13.1 (2.2)	12.8 (2.0)	13.5 (2.4)	0.065
Blood glucose level, mg/dL	96.2 (12.7)	95.9 (12.2)	96.4 (13.2)	0.844
Hypertension, *n* (%)	42 (35.9)	24 (40.7)	18 (31.0)	0.277
Cardiac disease, *n* (%)	7 (6.0)	4 (6.8)	3 (5.2)	0.714
Diabetes mellitus, *n* (%)	10 (8.5)	6 (10.2)	4 (6.9)	0.527
Arthritis, *n* (%)	16 (13.7)	9 (15.3)	7 (12.1)	0.616
Five or more medications, *n* (%)	20 (17.1)	11 (18.6)	9 (15.5)	0.653
Low frequency of going outdoors, *n* (%)	17 (14.8)	11 (20.3)	5 (8.9)	0.115
One or more falls in previous year, *n* (%)	22 (81.2)	14 (23.7)	8 (13.8)	0.169
Poor subjective health, *n* (%)	19 (16.2)	7 (11.9)	12 (20.7)	0.196
TMIG-IC (max, 13)	12.5 (0.8)	12.5 (0.8)	12.4 (0.8)	0.682
GDS (max, 15)	2.6 (2.4)	3.3 (2.5)	1.9 (2.1)	0.001
Anxiety (max, 30)	14.0 (6.0)	17.1 (5.5)	10.8 (4.8)	<0.001
Neuroticism (max, 3)	0.7 (0.8)	0.5 (0.6)	0.8 (0.9)	0.074
RSES (max, 40)	4.3 (1.5)	3.9 (1.5)	4.7 (1.3)	0.001
MMSE (max, 30)	29.3 (0.9)	29.2 (1.0)	29.4 (0.8)	0.155
Grip strength, kg	23.7 (6.9)	22.1 (6.1)	25.4 (7.3)	0.008
Usual gait speed, m/s	1.49 (0.33)	1.48 (0.25)	1.51 (0.41)	0.710
Fast gait speed, m/s	2.32 (0.45)	2.26 (0.43)	2.37 (0.47)	0.183
TUG, s	5.28 (1.02)	5.35 (0.93)	5.22 (1.11)	0.632
One-leg standing test, s	44.8 (19.8)	44.8 (20.2)	44.8 (19.4)	0.999

*Note: FoF, fear of falling; BMI, body-mass index; TMIG-IC, Tokyo Metropolitan Institute of Gerontology-Index of Competence; max, maximum; GDS, Geriatric Depression Scale; RSES, Rosenberg Self-Esteem Scale; MMSE, Mini-Mental State Examination; TUG, Timed Up and Go test*.

**Figure 1:**
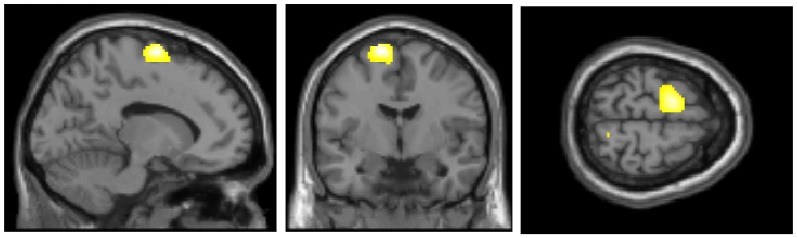
Regions of significantly decreased glucose metabolism in the Fear of Falling (FoF) group compared with the non-FoF group in the baseline assessment. The significance level for the correlated clusters was set at a voxel-level significance of *p* < 0.001 (uncorrected) combined with cluster-level information of *p* < 0.05 (family-wise error corrected).

Figure 1 presents the regions of significantly decreased cerebral glucose metabolism in the FoF group compared with the non-FoF group. After adjusting for age, gender, BMI and blood glucose level, the FoF group showed significantly decreased glucose metabolism in the left superior frontal gyrus (SMA or BA6). These results remained significant after adjusting for years of education, GDS score, anxiety, neuroticism, RSES and grip strength (Talairach Coordinates: *Z* = −13, *Y* = −7, *Z* = 63, *K* = 563, *T*-value = 5.03).

At the follow-up assessment, 19 participants had newly developed FoF (21.5% of the participants with no FoF at baseline). Table 2 presents a comparison of the baseline data for the D-FoF group with the N-FoF group. The D-FoF group showed significantly higher percentages of participants who have hypertension and arthritis, take five or more medications and had a fall in the previous year (with marginal significance, *p* < 0.054), slower gait speed under fast conditions (with marginal significance, *p* < 0.088), and longer TUG time (with marginal significance, *p* < 0.071) than those in the N-FoF group.

**Table 2:** Characteristics and differences of measurements of the participants with newly developed FoF (D-FoF) and those who did not report FoF (N-FoF) at follow-up assessment.

Baseline Characteristic, mean (SD)	D-FoF	N-FoF	*p*-value
	(*n* = 19)	(*n* = 39)	
Female, *n* (%)	15 (78.9)	25 (64.1)	0.251
Age	76.1 (4.8)	73.9 (5.0)	0.112
BMI	22.7 (3.0)	22.2 (3.0)	0.593
Years of education	13.5 (2.1)	13.5 (2.5)	0.984
Blood glucose level, mg/dl	95.5 (18.3)	96.8 (10.2)	0.735
Hypertension, *n* (%)	10 (52.6)	8 (20.5)	0.013
Cardiac disease, *n* (%)	1 (5.3)	2 (5.1)	0.983
Diabetes mellitus, *n* (%)	2 (10.5)	2 (5.1)	0.446
Arthritis, *n* (%)	6 (31.6)	1 (2.6)	0.001
Five plus medications, *n* (%)	6 (31.6)	3 (7.7)	0.018
Low frequency of going outdoors, *n* (%)	2 (10.5)	3 (7.7)	0.718
One or more falls in previous year, *n* (%)	5 (26.3)	3 (7.7)	0.054
Poor subjective health, *n* (%)	4 (21.1)	8 (20.5)	0.962
TMIG-IC (max 13)	12.5 (0.7)	12.4 (0.9)	0.541
GDS (max 15)	1.3 (1.7)	2.1 (2.2)	0.167
Anxiety (max 30)	9.4 (5.1)	11.5 (4.6)	0.121
Neuroticism (max 3)	0.9 (0.9)	0.7 (0.9)	0.477
RSES (max 40)	5.1 (0.9)	4.6 (1.4)	0.136
MMSE (max 30)	26.7 (1.6)	26.3 (2.4)	0.476
Grip strength, kg	22.1 (6.1)	25.4 (7.3)	0.526
Usual gait speed, m/s	1.48 (0.16)	1.55 (0.30)	0.302
Fast gait speed, m/s	2.26 (0.43)	2.37 (0.47)	0.088
TUG, s	5.64 (1.44)	5.07 (0.92)	0.071
One-leg standing test, s	44.8 (20.2)	44.8 (19.4)	0.835

*Note: FoF, fear of falling; BMI, body-mass index; TMIG-IC, Tokyo Metropolitan Institute of Gerontology-Index of Competence; max, maximum; GDS, Geriatric Depression Scale; RSES, Rosenberg Self-Esteem Scale; MMSE, Mini-Mental State Examination; TUG, Timed Up and Go test*.

**Figure 2:**
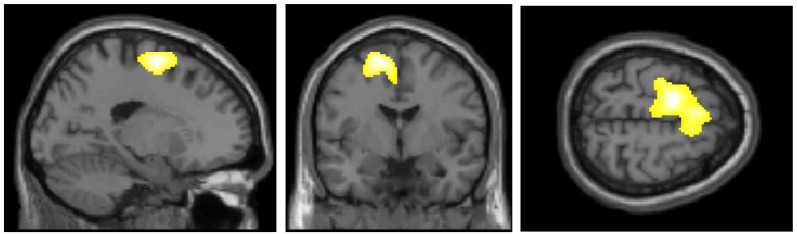
Regions of significantly decreased glucose metabolism in the newly developed FoF (D-FoF) group compared with the who did not report FoF (N-FoF) group at follow-up assessment. The significance level for the correlated clusters was set at a voxel-level significance of *p* < 0.001 (uncorrected) combined with cluster-level information of *p* < 0.05 (family-wise error corrected).

Figure 2 presents the regions of significantly decreased cerebral glucose metabolism in the D-FoF group compared with the N-FoF group. Of the 58 participants without FoF at baseline, a two-sample *t*-test showed significantly decreased glucose metabolism in the left middle frontal gyrus (BA6), which was independent of age, gender, BMI, blood glucose level, presence of hypertension and arthritis, number of medications, fall history, gait speed under fast conditions and TUG (Talairach Coordinates: *Z* = −17, *Y* = −6, *Z* = 58, *K* = 2342, *T*-value = 4.51).

The adjusted logistic regression analysis using aforementioned covariates showed that the decreased normalized regional cerebral glucose metabolism in the left SMA was an independent predictor of the future development of FoF (Odds Ratio, 1.03; 95% Confidence Interval, 1.01–1.06; *p* = 0.015). This association remained significant after adjusting for GDS, anxiety, neuroticism, and RSES (Odds Ratio, 1.03; 95% Confidence Interval, 1.01–1.06; *p* = 0.030).

## Discussion

In this cohort of older adults free of impending physical and cognitive dysfunction at baseline, participants with FoF showed significantly decreased glucose metabolism in the SMA, which correspond to left superior frontal gyrus. Longitudinally, participants without FoF at baseline but having decreased glucose metabolism in the left superior frontal gyrus were more likely to develop FoF, independent of psychiatric symptoms and physical functioning at the baseline. Our findings suggest that low functionality of the SMA, which is critical for motor planning and coordination, may contribute to FoF. To the best of authors’ knowledge, our results provide the first evidence of a potential mechanism underlying the relationship between aging brain and new-onset of FoF.

### Possible Association of SMA and Gait Control with FoF

Any cortical deficits or reduced metabolism in the regions related to gait control may result in FoF because deficits in gait control can predispose us to a severe accident, which is the main cause of developing FoF. Numerous studies have indicated that the SMA is involved in motor control (Harada et al., 2009; Iseki et al., 2010). For instance, Harada et al. (2009) used functional near-infrared spectroscopy to demonstrate increased oxygenated hemoglobin in the SMA during walking in older adults and correlation of its activation with locomotor speed and cadence. This finding suggests that the SMA controls gait by regulating stride frequency (i.e., cadence). Considering that older adults with FoF tend to show a lower stride frequency (Rochat et al., 2010), low functionality of the SMA might affect onset of FoF through deficit in gait control, such as lower performance of stride frequency.

This speculation, the association of SMA and gait control with FoF, is also supported by the evidence that gait variability—a reflection of the inconsistency in the central motor control system’s ability to regulate gait and maintain a steady and stable walking pattern—is associated with having and developing FoF among older adults (Rochat et al., 2010; Ayoubi et al., 2014; Sawa et al., 2014). Although the underlying neural mechanism of the association between increased gait variability and FoF is still unclear, one experimental study revealed an association between the amount of activity seen in the SMA and the amount of gait variability (Kurz et al., 2012), suggesting that the SMA may play a prominent role in the subtle motor control. Since this previous finding does not directly explain why reduced neural activity in the SMA is associated with new-onset of FoF, future studies are needed to examine interrelationship between change in SMA activity, gait performance (e.g., stride frequency and gait variability), and FoF.

### Possible Association of SMA and Motor Planning with FoF

We found that the relationship between glucose metabolism in the SMA and FoF was seen in the left hemisphere only. This result is in agreement with the concept that deficit in motor planning is associated with the expression of FoF. The left SMA plays an important role in the elaboration/preparation of motor behavior (Ackermann et al., 1996; White et al., 2013). Previous studies have shown that older adults with FoF face environmental challenges, and they are not able to select the appropriate strategy for visual searching and anticipatory postural adjustments to overcome these fall hazards (Uemura et al., 2012; Young and Mark Williams, 2015). More recently, Sakurai et al. (2017a) have shown that older adults with FoF cannot accurately imagine their gait performance, and a significant relationship exists between deficits in gait-related motor imagery ability, which is the ability to mentally simulate an action without its actual execution, and FoF. Motor imagery likely corresponds to the activation of the neural representations of motor planning in the frontoparietal network, including the SMA (Jeannerod and Decety, 1995). These findings suggest that reduced activity in the SMA, particularly in the left hemisphere, contributes to a functional decline in motor planning and the expression of FoF.

### Differences in the Findings between a Previous Study and the Present Study

In contrast to a prior study (Tuerk et al., 2016), we did not find any interactions of the psychological factors in the relationship between cerebral glucose metabolic change and FoF. One possible reason for this inconsistency is the different methods that were used to measure FoF. The previous study used the Falls Efficacy Scale-International (FES-I) to assess the participants’ concern about falling in various situations in ADL, such as cleaning the house, shopping and walking on uneven surfaces, whereas a dichotomized question was used in the present study. Psychological factors are likely to be strongly associated with the results of the FES-I, which reflect the different levels of FoF in various circumstances. Furthermore, we used different measures to examine participants’ psychological characteristics from the previous study. If we had measured the participants’ levels of concern about falling with the FES-I and used same measures for examining psychological factors, we might have found interesting results for the effects of psychological factors on the relationship between reduced cerebral metabolism and FoF.

### Limitations

Some limitations in our study need to be considered. First, as discussed above, although a dichotomized question for assessing FoF is widely used, there is limited evidence about the measurement properties of this single-item measure and this approach was limited to measuring different levels of FoF in various circumstances (Jørstad et al., 2005). Second, we used one question of the 36-item Short-Form Health survey to examine neurotic personality. However, since the question is not a question developed for assessment of neurotic personality, it might be insufficient to confirm such personality. Third, we included a relatively small number of participants which may affect the power to find significant additional associations between change in cerebral glucose metabolism and FoF. Fourth, our participants were all high functioning older adults free of impending physical and cognitive dysfunction, and, consequently, our results are only generable to high functioning populations. Finally, one-time assessment of FDG-PET during 2 years of follow-up may not be enough to detect the causal relationship between changes in cerebral glucose metabolism with aging and new-onset of FoF. Further studies including multiple follow-up assessments are needed to confirm the results of the present study in a larger sample with more detailed measures of FoF.

## Conclusion

Lower cerebral glucose metabolism in the SMA was associated with the development of FoF in our cohort of high-functioning older adults. This suggests that functional impairments in motor planning and motor control might be an early phenomenon in the development of new-onset FoF and precede significant impairments in physical function. Our findings provide mechanistic evidence of a potential mechanism underlying the relationship between the aging brain and FoF.

## Author Contributions

RS, YF, KK, MM-O and KI: manuscript preparation and editing; data analysis and interpretation. RS, YF, MY, HS and KI: acquiring data. RS, YF, MY, HS, KK, MM-O and KI: study design. RS, YF, KK and KI: study funding.

## Conflict of Interest Statement

The authors declare that the research was conducted in the absence of any commercial or financial relationships that could be construed as a potential conflict of interest.
